# A safety and effectiveness evaluation of RefluxStop in the treatment of acid reflux comparing large and small hiatal hernia groups: results from 99 patients in Switzerland with up to 4-years follow-up

**DOI:** 10.1007/s10029-025-03339-2

**Published:** 2025-05-03

**Authors:** Yves Borbély, Dino Kroell, Sarah Gerber, Yannick Fringeli, Ioannis Linas, Joerg Zehetner

**Affiliations:** 1 Department of Visceral Surgery and Medicine, Inselspital, Bern University Hospital, Bern, Switzerland; 2 Department of Visceral Surgery, Hirslanden Clinic Beau-Site, Bern, Switzerland; 3 Department of Gastroenterology, Hirslanden Clinic Beau-Site, Bern, Switzerland

**Keywords:** Gastroesophageal reflux disease (GERD), GERD-HRQL, Dysmotility, Motility disorder, Hiatal hernia, Large hiatal hernia, Antireflux surgery, Adverse event, Adverse device effect, Dysphagia, Gas-bloating, Surgical option

## Abstract

**Background:**

Standard-of-care surgical treatments for gastroesophageal reflux disease (GERD), with large hiatal hernia (HH), result in a reoperation rate of up to 50% at 5 years. RefluxStop, acting as a mechanical stop without encircling the food passageway, offers a novel approach to treat large HH patients. This study assesses the safety and efficacy of RefluxStop surgery comparing large and small HH groups followed for up to 4 years.

**Methods:**

Two cohorts were retrospectively analyzed in a combined investigator-initiated study evaluating safety outcomes of RefluxStop in severe GERD subjects, comparing concomitant small (≤3 cm) and large HH (4–10 cm) in Switzerland. Primary outcomes were procedure-related adverse events (AEs/ADEs). The secondary outcome was improvement in GERD-HRQL score.

**Results:**

Ninety-nine subjects underwent the RefluxStop surgical procedure, whereof 50 subjects had small (≤3 cm) and 49 subjects had large HH (4–10 cm). One surgeon at each site operated on both small and large hernia patients. No significant difference in AEs between patients with small and large HH was shown. At 1-year follow-up, subjects in both groups experienced statistically significant improvements in median (IQR) GERD-HRQL score of 93.8% (81.8%; 98.7%) for those with large HH and 85.7% (76.5%; 92.3%) for those with small HH.

**Conclusion:**

RefluxStop surgery for GERD effectively treats patients with large HH that currently have no optimal treatment options, while showing significantly improved results for up to 4 years. Furthermore, RefluxStop provides equally favorable results and a robust low risk profile for subjects with either concomitant small (*n* = 49) and large (*n* = 50) HH.

## Introduction

Gastroesophageal reflux disease (GERD) is a serious ailment due to the associated progressive complications, including significant mortality due to subsequent esophageal adenocarcinoma via Barrett’s esophagus. For instance, in the United States (US) and European Union (EU) alone, about 48,000 persons die from esophageal adenocarcinoma with most of them attributable to acid reflux [1–6]. Repeated acid exposure leads to tissue damage (i.e., esophagitis) and precancerous changes (i.e., Barrett’s esophagus) that fulminate to carcinoma. Barrett’s esophagus occurs in up to 20% of GERD patients [4–6], with subsequent progression to esophageal carcinoma at an average incidence rate of 0.6% [7]. Unfortunately, about 85% of those suffering from esophageal cancer succumb to the illness [3, 7]. These considerations merit significant attention when bearing in mind that GERD affects nearly 20% of the US population on a weekly basis [8].

Currently, first-line management of reflux disease is proton pump inhibitor (PPI)-based therapy. However, PPIs do not directly address the cause of reflux itself, but instead only reduce the acidity of gastric contents flowing back into the esophagus, without reducing the frequency of reflux episodes, increasing lower esophageal sphincter (LES) tone, or reducing transient LES relaxations [9–12]. Moreover, up to 59% of patients continue to experience intermittent heartburn while on PPI therapy [13] and 30–40% have complete irresponsiveness to drug therapy, as evidenced by persistent GERD symptoms with pH-measurable reflux episodes [11]. More specifically, it has been reported that almost 40% of daily PPI users continue to have a pathologic result on 24-hour pH monitoring [14]. Furthermore, many studies report long-term risks of PPI use, such as a study by Yan Xie et al. on excess mortality [2].

Several surgical options currently exist as an alternative to PPI-based medical therapy in the management of GERD. However, these antireflux surgeries, such as Nissen fundoplication (i.e., gold-standard) and magnetic sphincter augmentation (MSA) via the LINX system, use mechanisms of action that encircle or augment the lower esophagus, part of the food passageway [15, 16]. The challenge with this approach is effectively treating acid reflux without incurring serious swallowing difficulties and related side effects.

In cases of large hiatal hernia (HH) ≥3 cm size, there is a unique challenge in the management of reflux patients. More recently developed antireflux procedures are not approved, recommended, or evaluated as an effective treatment [17–19]. These include MSA, endoscopic transoral incisionless fundoplication, and the Medigus Ultrasonic Surgical Endostapler (MUSE), thus providing a limited repertoire of options to manage disease. For such patients, the currently available surgical treatments often do not provide favorable outcomes with a high rate of reherniation independent of the use of mesh, with up to 55% reccurrence at 5 years as per a randomized clinical trial by Watson et al. from 2020 [20]. A shorter study assessing hernia recurrence after antireflux surgery, for temporal comparison, reports a 15% reherniation rate with 20-month follow-up [21]. Since large HH is associated with a high rate of recurrence after antireflux management, it is clear that innovative surgical strategies are necessary to mitigate this substantial unmet need that is rapidly growing around the world.

A novel method, the RefluxStop™ (Implantica, Zug, Switzerland) procedure, addresses this crucial treatment gap through its distinctive therapeutic principle that aims to prevent reherniation in large HH cases, by not encircling the food conduit and applying circumferential pressure. The approach of RefluxStop is intended to prevent gastroesophageal junction mobility superiorly into the chest cavity while maintaining dynamic flexibility during respiration and alimentation, acting as a mechanical anchor in the abdomen. Implantation of the RefluxStop device ensures an appropriate distance between the LES and diaphragm, thereby preventing acid reflux. Furthermore, the procedure aims to correct all aspects of the antireflux barrier through realignment of the angle of His, treating reflux disease by restoring the normal anatomy and physiology of the gastroesophageal junction without incurring complications associated with augmentation of the food passageway.

The purpose of this study is to assess the safety results of the RefluxStop device in treating patients with reflux disease and concomitant HH comparing two groups, small and large HH, to better understand the role of this novel treatment option in the reflux treatment landscape. We present the safety and effectiveness outcomes of RefluxStop surgery in Swiss GERD patients with severe disease and concomitant small or large HH for up to 4 years. The combined cohorts presented herein are, from a clinical perspective, well-suited for comparison of results in small and large HH subgroups since patients were operated on by a solitary, surgeon at each center.

## The RefluxStop procedure

**Fig. 1:**
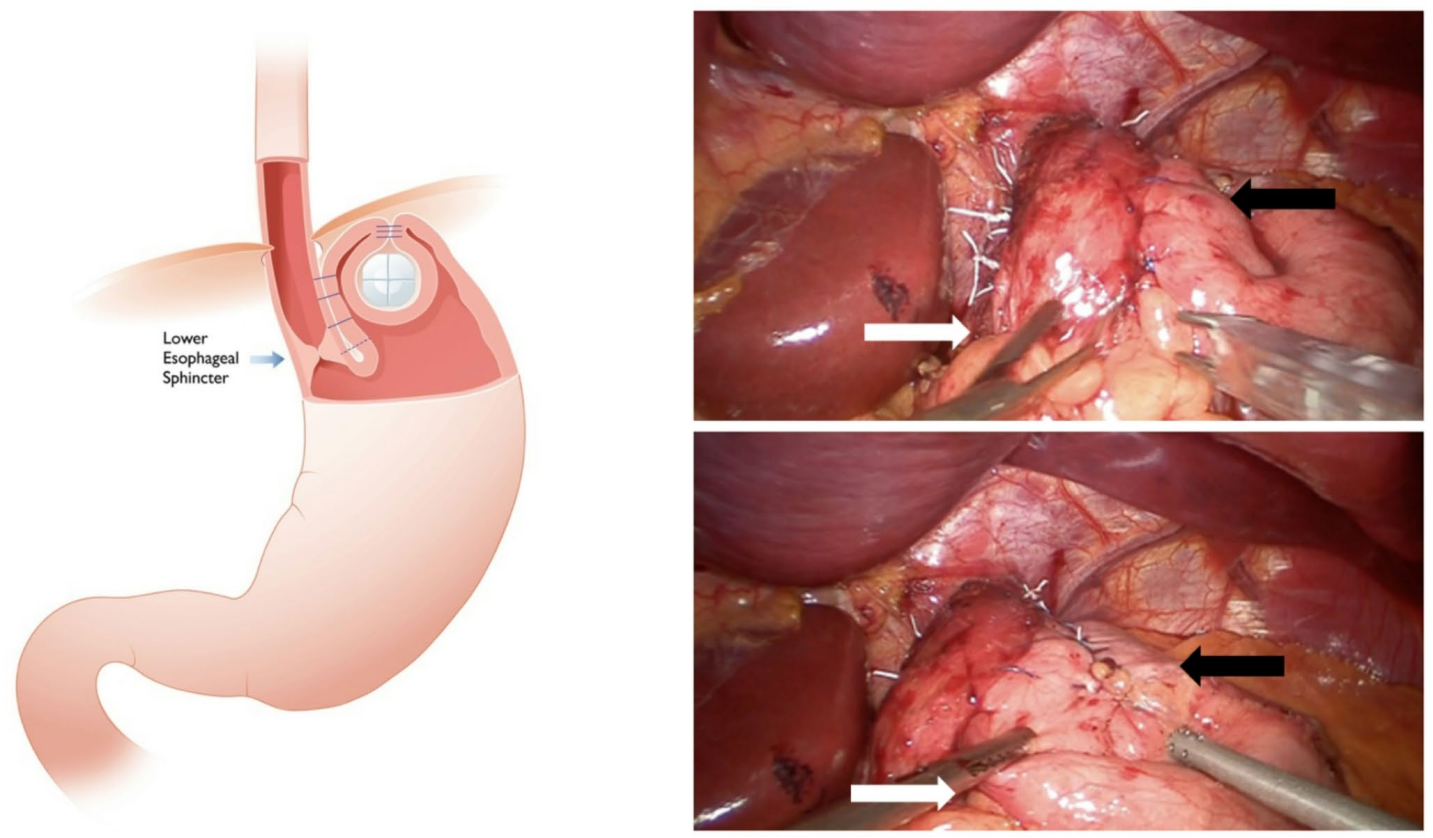
The RefluxStop implant **(A)** placed in a fundic pouch to restore and maintain the newly constructed antireflux barrier, and **(B)** intraoperative images of the completed RefluxStop procedure. White arrows denote the reconstructed acute angle of His and black arrows show the relative position of the bottom of the device (invaginated in the fundic pouch). *LES*,* lower esophageal sphincter*

The RefluxStop procedure uses a novel treatment principle that is intended to treat reflux disease by restoring the antireflux barrier and maintaining regional anatomical and physiological stability, even during dynamic processes like respiration. The device is composed of medical-grade silicone that is considered to be inactive. The thesis behind its mechanism of action includes one important function that is designed to reduce reherniation in large HH subjects: Fundus reinforcement with RefluxStop is thought to act like a mechanical stop, maintaining the LES inferiorly in the abdomen. Esophageal dissection high up in the mediastinum, esophagogastroplication (90–110°), and placement of the RefluxStop implant in an invagination on the outside of the gastric fundus using a dedicated deployment tool, provides anchoring and stability of the newly restored antireflux physiology and anatomy. Ultimately, the result of RefluxStop surgery resembles the depiction in Fig. 1.

## Methods

### Study design and objectives

A retrospective chart review was conducted in two investigator-initiated studies at two tertiary level hospitals assessing the safety outcomes of the RefluxStop procedure in GERD subjects with concomitant HH comparing small hernia (≤3 cm) and large hernia (4–10 cm) in Switzerland. Each independent analysis assumed the clinical perspective of comparing safety outcomes for small and large hernia, rigorously following all adverse events (AEs), with one surgeon operating on both small and large hernia subjects at each site.

### Baseline investigations

Subjects underwent routine pre- and postoperative evaluations for antireflux surgery, including:

- GERD Health-Related Quality of Life (GERD-HRQL) questionnaire.
- Upper GI endoscopy.
- Simplified barium swallow.
- 24-hour pH impedance monitoring (together with manometry). In one center, large hernia (> 3 cm) patients and patients with esophagitis grade C and D, and those with Barrett’s esophagus did not undergo pH testing.
- Personal demographics and medical data, including imaging data, directly relevant to subject’s therapy for GERD.

### Clinical outcomes

The primary endpoints of this analysis were incidence of adverse device effects (ADEs) and procedure-related AEs, which were followed at hospital stay, 6 weeks and 6 months after surgery, and annually thereafter; unscheduled visits were also included. These were further delineated into intra- and postoperative AEs. The secondary endpoint was improvement in total GERD-HRQL score from baseline to follow-up, as an indicator of treatment effectiveness and to add additional context to the safety outcomes. The GERD-HRQL score was measured at baseline and at least annually at 1- to 4-year follow-ups.

### Ethics

This study was conducted in compliance with the Declaration of Helsinki. Before implementation of this study, the investigation plan and proposed subject information were reviewed by the Regional Ethics Committee (EX) for the Canton of Bern (Kantonale Ethikkommission Bern). A signed and dated statement that the investigation plan and patient information were approved by the ethics committee and provided to the sponsor investigator before study initiation. All subjects provided informed consent for the use of their clinical data for research purposes.

### Data protection and quality assurance

Data handling fulfills all requirements of the Human Research Act. Study-related subject data was collected in a coded manner and stored in a locked server-room. Data extracted from medical records were checked for completeness and plausibility (e.g., inconsistencies and out-of-range values).

### Subject population

#### Inclusion criteria

- ≥18 years of age.
- Documented GERD present > 6 months.
- Presence of hiatal hernia of various sizes.
- Endoscopy-verified esophagitis or 24-hour pH monitoring-proven GERD (i.e., defined as pH < 4 for > 4.5% of the total time) off PPI therapy for at least 7 days prior to testing.
- In one center, subjects with large hiatal hernia (> 3 cm), Barrett’s esophagus, or reflux esophagitis Grade C or D (Los Angeles classification) qualified for surgery without pH testing.

#### Exclusion criteria

- No consent to perform procedure.
- Patients < 18 years of age.
- Known presence of delayed gastric emptying, if no other cause for acid reflux could be diagnosed.
- History of bariatric surgery, wherein the gastric fundus has been extirpated.
- Female patients who are pregnant or nursing.
- Known sensitivity or allergies to silicone materials.
- Intraoperative findings that may result in unfavorable conduct of procedure.

### Concomitant medications/treatments

Subjects were evaluated using gastroscopy and functional diagnostics preoperatively and at annual follow-ups. GERD-HRQL scores were documented preoperatively and at annual follow-ups. Subjects were generally discharged on postoperative day 1. Subjects’ intakes were restricted to liquid and pulpy food during the first 10 days postoperatively. On postoperative day 10, sutures were removed.

### Follow-up

Routine follow-up took place immediately after surgery, at 6 weeks, 6 months, and 12 months primarily for safety, as well as annually thereafter up to 4 years postoperatively.

### Statistical design

Descriptive statistics were used to present the safety outcomes and descriptive analyses were used to summarize the analysis of GERD-HRQL score improvement. For descriptive analyses, parametric data were presented using minimum, maximum, median, quartiles (IQR) (Q25; Q75), mean, and standard deviation (SD). Categorical parameters were presented using absolute counts and percentages. Distribution of parametric data was inspected by box plots. All available data were included and summarized in the analyses. Missing data were not substituted but handled as ‘missing’ in the statistical evaluation.

## Results

### Patient characteristics

Two cohorts of subjects with concomitant HH were treated by RefluxStop in Switzerland. A total of 102 subjects were operated on between April 2019 and November 2022, whereof 99 of 102 subjects accepted study participation. Moreover, 50 patients had small hernia (≤3 cm) and 49 had large hernia (4–10 cm); no patients had HH between 3 and 4 cm in size. Hiatal hernia size was determined by measurements from endoscopic, radiologic, and manometric studies (i.e., not during surgery) where the largest measurement was used for this report. Size was typically measured from the Z-line and crura on endoscopy, the same estimation used for swallow studies and manometry. Hiatal surface area measurement was not used as it is not standard practice at our institutions in Switzerland and we had followed the standard measurement procedure that is consistent with our clinical practice and of several other European institutions. Since consensus is lacking in defining the thresholds for small and large hiatal hernia [22], categorization in this study followed expert opinion, where size is typically denoted as follows: small (1–2 cm); large (3–8 cm); and very large (> 8 cm) or “upside-down stomach”. Cohort 1 included 45 operative cases (43 accepted study participation). Cohort 2 included 57 operative cases (56 accepted study participation). Baseline patient characteristics are outlined based on small (≤3 cm) and large (4–10 cm) HH designation in Table 1.

**Table 1:** Subject characteristics based on small (≤3 cm) and large (4–10 cm) HH groups of the study

Characteristic	Small hernia (*n* = 50)	Large hernia (*n* = 49)
Age (SD), years	50.1 (15.1)	58.4 (15.1)
Sex, n (%)		
Male	27 (51.9%)	25 (48.1%)
Female	28 (56%)	22 (44%)
BMI (SD), kg/m^2^	26.8 (4.1)	26.9 (4.3)
Esophageal disorders, n (%)		
Barrett’s esophagus	14/31 (45.2%)**	17/39 (43.6%)**
Dysphagia (preoperative)	14 (26.9%)	21 (42%)
Esophageal dysmotility	27 (51.9%)	39 (78%)
Gas bloating	12/31 (38.7%)	N/A
Hiatal hernia size, cm		
Median (Q1; Q3) (max-min)	2 (1; 3)	5 (4; 5) (4–10 cm)
Mean (SD)	2.1 (0.9)	5.0 (1.3)

BMI, body mass index; N/A, not available; SD, standard deviation

** Barrett’s esophagus– Data available on 31small and 39 large hernia patients

### Primary outcome: ADEs and procedure-related AEs

All AEs that may be procedure- or device-related are summarized in Tables 2 and 3. Identification of AEs was based on GERD-HRQL score and patient-initiated complaints were also recorded. Overall, the RefluxStop procedure provided excellent and equivalent safety outcomes in both small and large HH patient groups for 4 years. These tables provide a side-by-side comparison of safety outcomes in small and large hiatal hernia subjects. Adverse outcomes are presented with SAEs listed first, followed by AEs, since urgency/severity is most clinically relevant. The severity of an outcome is denoted by mild, moderate, or severe. Furthermore, outcomes are categorized as procedure- or device-related, where applicable, with the time of occurrence and resolution/recovery status indicated. Altogether, most SAE/AEs were short-term and completely resolved. The small hernia group (*n* = 50) experienced one SAE and 17 AEs (of which only four AEs were procedure-related), where all procedure-related events resulted in resolution/recovery. The large hernia group (*n* = 49) experienced two SAEs and 12 AEs, where only one was unresolved and one status was unknown.

**Table 2:** AEs in subjects with small hiatal hernia (≤3 cm) from both cohorts (*n* = 50)

Adverse event	AE or SAE	Relation to device or procedure	Severity	Time period	Recovered/Not resolved
Esophageal perforation noticed directly during surgery (accident by trainee surgeon), esophageal stent placement & removal, rehospitalization also for stent explant^1^	SAE (*n* = 1)	Procedure	Moderate	During procedure	Recovered
Liver retractor causing small liver laceration (frequent low impact AE)^1^	AE (*n* = 1)	Procedure	Mild	During procedure	Recovered
Hernia at a trocar location which was surgically repaired	AE (*n* = 1)	Procedure	Moderate	0–30 d	Resolved
Wound dehiscence with skin necrosis at 3 trocar sites iterative hospital visits	AE (*n* = 1)	Procedure	Moderate	0–30 d	Recovered
Balloon dilatation due to persistent dysphagia– successful outcome (*n* = 2)	AE (*n* = 2)	Procedure	Moderate × 2	1–6 mo	Resolved
Acid taste intraoral, unpleasant	AE (*n* = 1)	Unlikely	Mild	1–6 mo	Recovered
Pain, bloating	AE (*n* = 1)	Unlikely	Moderate	1–6 mo	Unknown
Dysphagia Mild remaining in two IEM patients	AE (*n* = 2)	Unlikely	Mild × 2	1–6 mo	Not resolved & unknown
Heartburn (*n* = 2), one with normal pH	AE (*n* = 2)	Unlikely	Mild	1–6 mo	Not resolved
			Moderate	6–12 mo	
Endoscopy exploration, for swallowing compliants from IEM patient. No significant stenosis however a one time not necessary balloon dilatation (*n* = 1)	AE (*n* = 1)	Unlikely	Mild	6–12 mo	Recovered
Gas-bloating symptoms not fully disappeared (*n* = 4)	AE (*n* = 4)	Unlikely	Mild × 4	6–12 mo (*n* = 2) 1– 6 mo (*n* = 2)	Unknown

AE, adverse event; SAE, serious adverse event

**Table 3:** AEs in subjects with large hiatal hernia (4–10 cm) from both cohorts (*n* = 49)

Adverse event	AE or SAE		Relation to device or procedure	Severity	Time period	Recovered/Not resolved
Hiatal hernia recurrence as consequence of severe vomiting from food poisoning (preop 8 cm HH)	SAE (*n* = 1)		Procedure	Moderate	1–6 mo	Resolved
Temporary fatigue due to pericardial effusion, treated successfully with drugs	SAE (*n* = 1)		Unrelated (or procedure)	Mild	0–30 d	Resolved
Laparoscopy switched to laparotomy due to adhesions and associated bleeding	AE (*n* = 1)		Procedure	Moderate	0–30 d	Resolved
Orbital emphysema & globus pharyngitis above jugulum	AE (*n* = 1)		Likely related to procedure	Mild	0–30 d	Recovered
Elevated CRP 6 h after procedure– spontaneously disappeared without consequences	AE (*n* = 1)		Unlikely	Mild	0–30 d	Recovered
Postop balloon dilatation due to dysphagia	AE (*n* = 1)		Procedure	Mild	1–6 mo	Resolved
Serom trocar, fistulation excluded by radiology	AE (*n* = 1)		Procedure	Moderate	1–6 mo	Recovered
Unnoticed early migration/penetration due to too tight sutured pouch as per the investigator– device spontaneously exited through anus, no symptoms	AE (*n* = 1)		Procedure	Mild	6–12 mo	No symptoms
Heartburn	AE (*n* = 1)		Unlikely	Mild	6–12 mo	Not resolved
Gas-bloating symptoms not fully disappeared (*n* = 4)	AE (*n* = 4)		Unlikely	Mild × 4	6–12 mo	Unknown
Redo to Toupet fundoplication on patient request. Patient with severe dysmotility and diversified symptoms– unchanged after Toupet	AE (*n* = 1)	Unlikely		Moderate	24–30 mo	Unchanged symptoms after Toupet fundoplication reoperation (caused by dysmotility, not acid reflux)

AE, adverse event; CRP, C-reactive protein; SAE, serious adverse event

#### In the large hernia group

One patient underwent reoperation due to a total collapse of HH repair from vomiting, caused by food poisoning. The fundus, with the device intact in its pouch, was repositioned and a new hiatal repair with mesh was performed. One patient had an unnoticed early penetration of the device into the gastric cavity that spontaneously and uneventfully exited the digestive tract through natural means (i.e., the device is designed in pieces). The surgeon believed it was caused by suturing of the fundic pouch too tightly, which is the predominant reason for such an early penetration; no action was required.

Another non-device-related side effect occurred in the large hernia group postoperatively: One case of pericardial effusion that was successfully managed conservatively. Dysphagia and gas-bloating were highly prevalent before surgery. In one of the centers, 49% of patients had dysphagia and most had gas-bloating, which in addition to large HH indicates a significantly difficult-to-treat group of patients. One IEM patient had persistent dysphagia from baseline and successfully underwent esophageal dilatation. One patient with severe dysmotility requested redo surgery with Toupet fundoplication, however, the patient’s symptoms remained unchanged and severe dysmotility was deemed the cause.

#### In the small hernia group

One (*n* = 1) subject experienced intraoperative esophageal perforation, that was repaired intraoperatively, and for precaution, the patient received an esophageal stent that was later removed.

One Hernia at a trocar position was surgically repaired. Another patient had skin necrosis at three trocar sites that occurred within 30 days postoperatively. As mentioned, this was a group of patients with severe disease and high frequencies of dysphagia and gas-bloating before surgery. Of all the patients with dysphagia preoperatively, two patients went through an endoscopic esophageal balloon dilatation. Gas-bloating also persisted in four patients out of the many IEM patients with gas-bloating preoperatively. A mild and small liver laceration by the liver retractor occurred; this is common in antireflux surgery.

### Secondary outcome: GERD-HRQL score

**Fig. 2:**
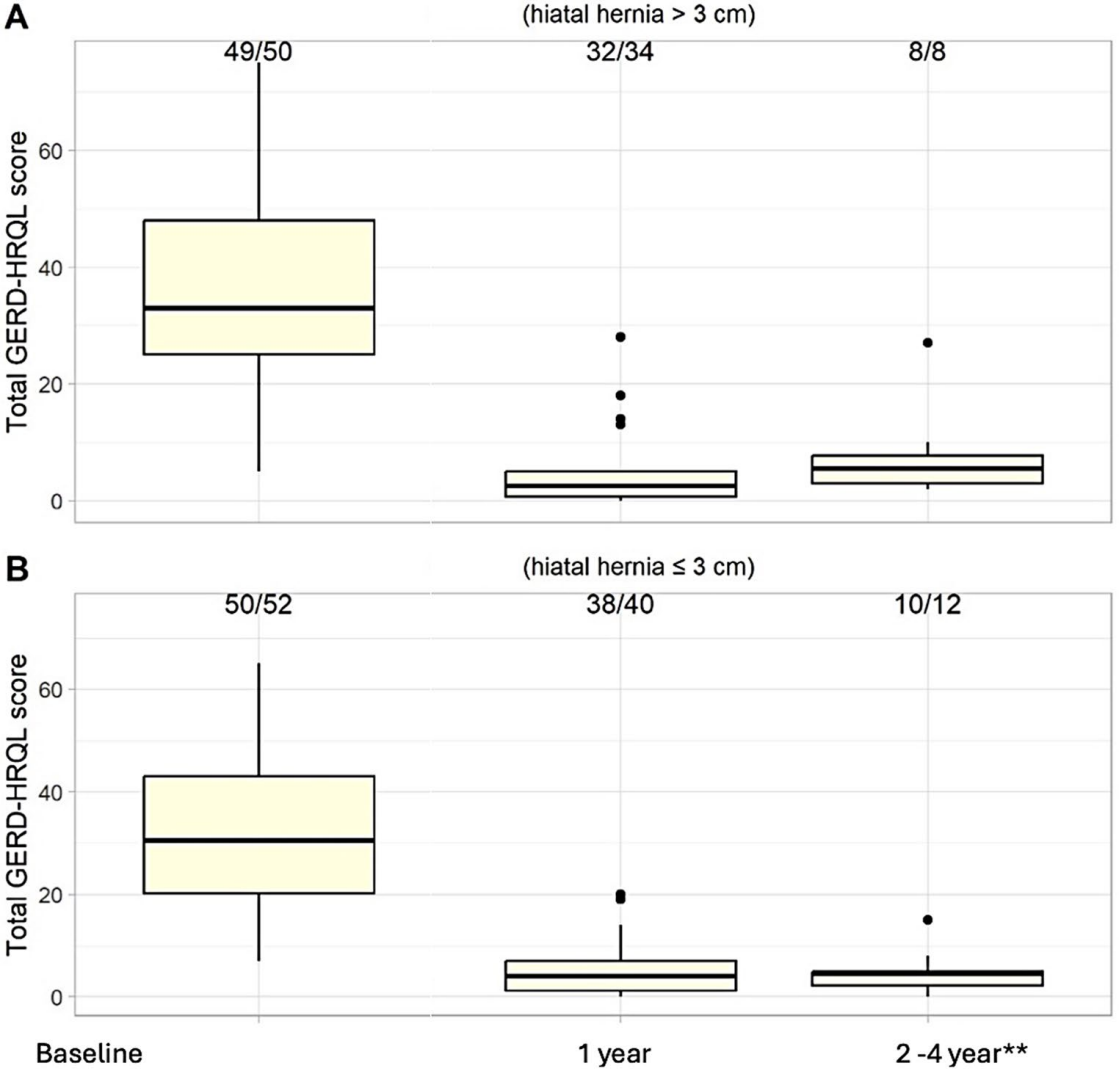
Total GERD-HRQL score over time in subjects operated on with **(A)** large HH of size 4 to 10 cm (*n* = 49) and **(B)** small HH of size ≤3 cm (*n* = 50) at two centers in Switzerland (i.e., University Inselspital and Hirslanden Clinic Beau-Site combined). In the large hernia group, total GERD-HRQL score improved by a median (Q25; Q75) of 93.8% (81.8%; 98.7%) (*n* = 32/34) and only two patients experienced < 50% improvement from baseline at year 1. In the small hernia group, total GERD-HRQL score improved by a median (Q25; Q75) of 85.7% (76.5%; 92.3%) (*n* = 38/40) and only two patients experienced < 50% improvement from baseline at year 1. In total, 93% of the patients had > 50% improvement of total GERD-HRQL score at year 1. Overall, the effectiveness of RefluxStop in treating reflux symptoms was comparable in subjects with concomitant small (≤3 cm) or large (4–10 cm) HH during the follow-up period thus far. GERD-HRQL, Gastroesophageal Reflux Disease Health-Related Quality of Life; HH, hiatal hernia. ** The 2-4-year follow-up includes the last score measured for all subjects that have reached 2-year follow-up or later after RefluxStop surgery, with the longest follow-up of 47 months, and the median (Q25; Q75) follow-up times: 27 (23; 35) months for subjects with hiatal hernia > 3 cm, and 25 (16; 27) months for subjects with hiatal hernias ≤3 cm

Total GERD-HRQL scores were included in this report to provide additional clinical context in conjunction with the primary safety outcomes. At 1-year follow-up of both cohorts, subjects experienced reductions in median (Q25; Q75) total GERD-HRQL score of 93.8% (81.8%; 98.7%) in the large HH group (*n* = 32/34) and 85.7% (76.5%; 92.3%) in the small HH group (*n* = 38/40). Overall, the effectiveness of RefluxStop in treating reflux symptoms was comparable in subjects with concomitant small (≤3 cm) or large (4–10 cm) HH during the follow-up period thus far (Fig. 2). This figure includes the last score measured for all subjects reaching 2-year (or longer) follow-up after RefluxStop surgery, with the longest follow-up of 47 months, and the median (Q25; Q75) follow-up times: 27 (23; 35) months for those with hiatal hernia > 3 cm; and 25 (16; 27) months for those with hiatal hernia ≤3 cm. In total, nine patients reached 3-year follow-up (whereof six had large hiatal hernia at baseline) and 93% of the patients had > 50% improvement of total GERD-HRQL score from baseline.

## Discussion

Large HH is thought to be an insurmountable risk factor for antireflux surgery with a reherniation frequency of up to 55% in the long term [16, 21, 23]. Moreover, patients with large HH have not been evaluated for many antireflux procedures, such as MSA with the LINX system [24], Stretta radiofrequency therapy [19], endoscopic transoral incisionless fundoplication [17], and MUSE [18], the latter two of which have a contraindication for HH larger than 3 cm in size. Thus, surgical treatment options for GERD patients with concomitant large HH are limited and outcomes are discouraging with the current standard of care, predominantly contingent on failure of hernia repair. For instance, a randomized trial by Watson et al. [20] from 2020 compared large hernia repair in three groups (i.e., without mesh, with non-resorbable mesh, and with resorbable mesh) with 5-year follow-up and found the highest recurrence rate (56%) in the group managed by resorbable mesh. Considering that this type of mesh repair is likely what surgeons utilize most in the current paradigm of antireflux surgery, the implications on long-term treatment success are concerning. The 20-month study by Armijo et al. [21] in 2019 reported follow-up more comparable to our study (i.e., up to 4 years ranging from 3 to 47 months in our study), in which a 15% hernia recurrence rate after standard-of-care antireflux surgery was appreciated.

Surgeons may have been reluctant to operate on large HH patients in the past due to high complication and treatment failure rates [25]. The RefluxStop device, on the other hand, has a design premise intended for treatment of this aruduously-managed patient group. For instance, as compared to the double-wall thickness of the cuff created during Nissen fundoplication, the RefluxStop device invaginated as part of a large fundic package that is adjacent to the diaphragmatic hiatus (as opposed to being in-line with it) seems to minimize the risk of gastroesophageal junction protrusion into the the chest cavity, logically speaking. According to Linnaus and colleauges [26], the recurrence of hiatal hernia has a high frequency on the ventral-left/upper-left aspect of the diaphragmatic hiatus. With RefluxStop surgery, a dorsal row of plication sutures results in reinforcement of the fundic package (presumably acting as a mechanical stop) located on the left-dorsal side, a region affected very little by recurrence according to Linnaus et al. [26]. Thus, one may argue that RefluxStop may reduce the reherniation rate in cases of large HH by interacting with a consistently preserved aspect of the diaphragmatic boundary. Moreover, there are other factors in the RefluxStop procedure that may reduce the likelihood of reherniation: dissection is performed extensively into the mediastinum; mobilization achieves a long intraabdominal esophagus; and reduced tension on the esophagus minimizes muscular contraction that may lead to reherniation. Taking this rationale to its logical conclusion, the only factor that may potentially lead to reherniation after RefluxStop surgery is total (or circumferential) collapse of hiatal repair, where the fundic package with the device invaginated in the center thereof (i.e., the device on its own is about 2.5 cm in size) is not large enough to prevent passage through the hiatal opening. In the study by Linnaus et al. [26], analysis of the position and extension of failed hiatus repair with median 5-year follow-up found a circumferential recurrence of 29%, the presupposed risk factor for reherniation after RefluxStop surgery. In such an instance, repositioning of the fundus followed by a new hiatus repair is possible, while leaving the RefluxStop implant untouched in its enclosed invagination pouch. As such, due to the design of RefluxStop as a mechanical stop intended to reduce hernia recurrence, it may have the potential to substantially reduce the recurrence rate of large HH in reflux surgical management, as indicated by these results.

RefluxStop treats the cause of acid reflux without encircling the food conduit and compounding swallowing difficulties, as is common in standard-of-care procedures [27–29]. The RefluxStop procedure aims to restore the angle of His, repair all three components of the antireflux barrier (i.e., the gastroesophageal flap valve, LES and its sling fibers, and the crural diaphragm) [30], re-institute optimal physiology and anatomy of the gastroesophageal junction, and maintain regional dynamic stability without invoking food passageway limitations. When the RefluxStop device was first implemented for use in real-world practice, large hiatal hernia patients were not specifically kept in mind and several surgeons used the implant based on clinical judgement and patient willingness, notably in severe cases (as shown in one of our centers particularly). The experience at our clinics prompted this investigation, to determine the relative treatment performance of this novel treatment between small and large hiatal hernia cases. This study showed comparable and excellent quality-of-life improvements in both small (median 85.7% improvement) and large (median 93.8% improvement) hernia groups, aligning well with the design rationale of RefluxStop in prevention of LES protrusion into the chest cavity. The relatively higher improvement in quality of life with large hernia patients is particularly notable since a larger proportion of large HH patients achieved long-term follow-up with better results as compared to the small hernia group. In total, nine patients reached 3-year follow-up, of which six had large hiatal hernia at baseline.

The patients operated on in this study belong to the most difficult-to-treat group pertaining to GERD, not only with 50% having large hernia (size 4–10 cm), but also a great number suffering from severe acid reflux. Approximately 44% (of the 70 patients tested) had Barrett’s esophagus, many with Los Angeles esophagitis grade C and D, and 35% had severe dysphagia prior to surgery. Moreover, most of these patients had gas-bloating preoperatively. The combination of large HH and dysphagia would be expected to result in suboptimal outcomes with high rates of reherniation and swallowing difficulties, affiliated with unfavorable quality-of-life (i.e., GERD-HRQL) scores. In view of the patient selection for this study, this report indicates that RefluxStop has a low risk profile and robustly treats acid reflux even in large HH patients, resulting in similar safety and effectiveness outcomes in those with small (≤3 cm) or large (4–10 cm) HH preoperatively.

This report provides important safety observations clinically relevant for implementation of this novel treatment option, particularly when considering the troublesome late side effects associated with fundoplication as a standard-of-care surgical option. In a recently published systematic literature review on Nissen fundoplication, results of nine studies found considerable rates of gas-bloating (52.7%), inability to vomit/belch (39.8%), dysphagia (28.9%), and heartburn or epigastric/sternal pain (27.0%) at 5 years after surgery [31]. These may be particularly accentuated in difficult-to-treat patients considering, inter alia, that those with large HH currently have unsatisfactory treatment options. Although not from the perspective of hiatal hernia patients, head-to-head study comparing RefluxStop to the standard of care, Nissen fundoplication, is currently underway and is expected to provide better resolution as to the role of this novel treatment option.

According to this analysis, RefluxStop implantation is well-tolerated even in these difficult-to-treat patients while also substantially improving patient quality of life, as documented by GERD-HRQL score. The GERD-HRQL score improved slightly more in the large HH group, albeit insignificantly as compared to the small HH group (i.e., median 94% improvement from baseline at 1 year in the large HH group compared to 86% in the small HH group), which may be attributed to more severe symptoms at baseline. Although surgical outcomes were basically equivalent between the large and small hernia groups of this study, one could not exclude the risk of a higher recurrence rate for large HH patients in the long term. However, as this study has indicated, the RefluxStop procedure is designed to, and has the potential to, substantially reduce such recurrence rates based on the logic described above. Thus, RefluxStop’s design and mechanism of action posit it as a promising treatment option in reflux patients with small and large HH alike. Longer follow-up and further randomized studies are in the pipeline.

### Strengths and limitations

A notable strength of this study is that it was performed at two separate centers, reducing the single-center bias. Furthermore, at each center, interoperator variation was eliminated at the individual cohort level since subjects were operated on by a single surgeon at each site, removing an uncertainty within each cohort that may have otherwise affected results. Between-cohort variability in subject demographics, hernia size, and proportion of subjects in categorical groups (i.e., small vs. large hernia) that resulted in similar safety and effectiveness outcomes, demonstrated the resilience of RefluxStop as a treatment option in difficult-to-treat patients with large HH. Rigorous documentation of intra- and postoperative AEs in conjunction with the low drop-out rates were other strengths of this study. Moreover, the study was conducted in real-world settings as per the clinical practice standard in Switzerland, reflecting real-life experience with more external validity. A limitation of this study is the relatively small sample size of each cohort, potentially augmenting the statistical influence of single, rare events. This study is not a randomized or prospective study, with the limitations thereof. Of course, a randomized trial would still have been preferred in this context, however, the study set-up with two centers improves the study data set.

## Conclusion

This study shows that the RefluxStop procedure is a promising treatment to manage reflux disease in both small (≤3 cm) or large (4–10 cm) HH patients. Despite patient selection including severe sufferers at baseline, with Barrett’s esophagus in 44% and severe dysphagia in approximately 35%, excellent safety results were reported for up to 4 years, with no significant difference in either safety or efficacy between the large (*n* = 49) and small hernia (*n* = 50) groups. Moreover, 93% of the patients had > 50% improvement of the GERD-HRQL score, demonstrating robust and successful treatment outcomes.

In the large hernia group, the total GERD-HRQL score improved by median 93.8% from baseline, and in the small hernia group, the total GERD-HRQL score improved by a median of 85.7% from baseline. Additional studies are underway and will continue to provide ancillary evidence on the safety and effectiveness outcomes of this unique and promising treatment option.
