# Correction to: A safety and effectiveness evaluation of RefluxStop in the treatment of acid reflux comparing large and small hiatal hernia groups: results from 99 patients in Switzerland with up to 4-years follow-up

**DOI:** 10.1007/s10029-025-03381-0

**Published:** 2025-06-12

**Authors:** Yves Borbély, Dino Kroell, Sarah Gerber, Yannick Fringeli, Ioannis Linas, Joerg Zehetner

**Affiliations:** 1 Department of Visceral Surgery and Medicine, Inselspital, Bern University Hospital, Bern, Switzerland; 2 Department of Visceral Surgery, Hirslanden Clinic Beau-Site, Bern, Switzerland; 3 Department of Gastroenterology, Hirslanden Clinic Beau-Site, Bern, Switzerland

**Correction to: Hernia**

10.1007/s10029-025-03339-2

In this article, the title was incorrectly given as 'A safety and effectiveness evaluation of refluxstop in the treatment of acid reflux comparing large and small hiatal hernia groups: results from 99 patients in Switzerland with up to 4-years follow-up' but should have been 'A safety and effectiveness evaluation of RefluxStop in the treatment of acid reflux comparing large and small hiatal hernia groups: results from 99 patients in Switzerland with up to 4-years follow-up'.

In the section 'The refluxstop procedure' in this article, the section heading 'The refluxstop procedure' should have read as 'The RefluxStop procedure'.

The original article has been corrected.

